# Cost-Effectiveness of Early Versus Standard Antiretroviral Therapy in HIV-Infected Adults in Haiti

**DOI:** 10.1371/journal.pmed.1001095

**Published:** 2011-09-20

**Authors:** Serena P. Koenig, Heejung Bang, Patrice Severe, Marc Antoine Jean Juste, Alex Ambroise, Alison Edwards, Jessica Hippolyte, Daniel W. Fitzgerald, Jolion McGreevy, Cynthia Riviere, Serge Marcelin, Rode Secours, Warren D. Johnson, Jean W. Pape, Bruce R. Schackman

**Affiliations:** 1 Division of Global Health Equity, Brigham and Women's Hospital, Boston, Massachusetts, United States of America; 2 Department of Public Health, Weill Cornell Medical College, New York, New York, United States of America; 3 Groupe Haitien d'Etude du Sarcome de Kaposi et des Infections Opportunistes (GHESKIO), Port au Prince, Haiti; 4 Department of Medicine, Weill Cornell Medical College, New York, New York, United States of America; Harvard School of Public Health, United States of America

## Abstract

**Background:**

In a randomized clinical trial of early versus standard antiretroviral therapy (ART) in HIV-infected adults with a CD4 cell count between 200 and 350 cells/mm^3^ in Haiti, early ART decreased mortality by 75%. We assessed the cost-effectiveness of early versus standard ART in this trial.

**Methods and Findings:**

Trial data included use of ART and other medications, laboratory tests, outpatient visits, radiographic studies, procedures, and hospital services. Medication, laboratory, radiograph, labor, and overhead costs were from the study clinic, and hospital and procedure costs were from local providers. We evaluated cost per year of life saved (YLS), including patient and caregiver costs, with a median of 21 months and maximum of 36 months of follow-up, and with costs and life expectancy discounted at 3% per annum. Between 2005 and 2008, 816 participants were enrolled and followed for a median of 21 months. Mean total costs per patient during the trial were US$1,381 for early ART and US$1,033 for standard ART. After excluding research-related laboratory tests without clinical benefit, costs were US$1,158 (early ART) and US$979 (standard ART). Early ART patients had higher mean costs for ART (US$398 versus US$81) but lower costs for non-ART medications, CD4 cell counts, clinically indicated tests, and radiographs (US$275 versus US$384). The cost-effectiveness ratio after a maximum of 3 years for early versus standard ART was US$3,975/YLS (95% CI US$2,129/YLS–US$9,979/YLS) including research-related tests, and US$2,050/YLS excluding research-related tests (95% CI US$722/YLS–US$5,537/YLS).

**Conclusions:**

Initiating ART in HIV-infected adults with a CD4 cell count between 200 and 350 cells/mm^3^ in Haiti, consistent with World Health Organization advice, was cost-effective (US$/YLS <3 times gross domestic product per capita) after a maximum of 3 years, after excluding research-related laboratory tests.

**Trial registration:**

ClinicalTrials.gov NCT00120510

*Please see later in the article for the Editors' Summary*

## Abstract 2

This cost-effectiveness study comparing early versus standard antiretroviral treatment (ART) for HIV, based on randomized clinical trial data from Haiti, reveals that the new WHO guidelines for early ART initiation can be cost-effective in resource-poor settings.

## Editors' Summary

**Background:**

AIDS has killed more than 25 million people since 1981, and about 33 million people (most of them living in low- and middle-income countries) are now infected with HIV, the virus that causes AIDS. HIV destroys immune system cells (including CD4 cells, a type of lymphocyte), leaving infected individuals susceptible to other infections. Early in the AIDS epidemic, most HIV-infected people died within 10 years of infection. Then, in 1996, highly active antiretroviral therapy (ART) became available and, for people living in affluent countries HIV/AIDS became a chronic condition. However, ART was extremely expensive and so a diagnosis of HIV infection remained a death sentence for people living in developing countries. In 2003, this situation was declared a global health emergency, and governments, international agencies, and funding bodies began to implement plans to increase ART coverage in developing countries. In 2009, more than a third of people in low- and middle-income countries who needed ART were receiving it, on the basis of guidelines that were in place at that time.

**Why Was This Study Done?:**

Until recently, the World Health Organization (WHO) recommended that all HIV-positive patients with CD4 cell count below 200/mm^3^ blood or an AIDS-defining illness such as Kaposi's sarcoma should be given ART. Then, in 2009, the CIPRA HT-001 randomized clinical trial, which was undertaken in Haiti, reported that patients who started ART when their CD4 cell count was between 200 and 350 cells/mm^3^ (“early ART”) had a higher survival rate than patients who started ART according to the WHO guidelines (“standard ART”). As a result, WHO now recommends that ART is started in HIV-infected people when their CD4 cell count falls below 350 cells/mm^3^. But is this new recommendation cost-effective? Do its benefits outweigh its costs? Policy-makers need to know the cost-effectiveness of interventions so that they can allocate their limited resources wisely. A medical intervention is generally considered cost-effective if it costs less than three times a country's per capita gross domestic product (GDP) per year of life saved (YLS). In this study, the researchers assess the cost-effectiveness of early versus standard ART in the CIPRA HT-001 trial.

**What Did the Researchers Do and Find?:**

The researchers used trial data on the use and costs of ART, other medications, laboratory tests, outpatient visits, radiography, procedures, and hospital services to evaluate the costs associated with early ART and standard ART among the 816 CIPRA HT-001 trial participants. The average total costs per patient after a maximum of 3 years treatment were US$1,381 for early ART and US$1,033 for standard ART. These figures dropped to US$1,158 and US$979, respectively, when the costs of research-related tests without clinical benefit were excluded. Patients who received early ART had higher average costs for ART but lower costs for other aspects of their treatment than patients who received standard ART. The incremental cost-effectiveness ratio after 3 years for early ART compared to standard ART was US$3,975/YLS if the costs of research-related tests were included in the calculation. That is, the cost of saving one year of life by starting ART early instead of when the CD4 cell count dropped below 200/mm^3^ was nearly US$4,000. Importantly, exclusion of the costs of research-related tests reduced the incremental cost-effectiveness ratio of early ART compared to standard ART to US$2,050/YLS.

**What Do These Findings Mean?:**

Because the Haitian GDP per capita is US$785, these findings suggest that, in Haiti, early ART is a cost-effective intervention over a 3-year period. That is, the incremental cost per year of life saved of early ART compared to standard ART after exclusion of research-related tests is less than three times Haiti's per capita GDP. The researchers note that their incremental cost-effectiveness ratios are likely to be conservative because they did not consider the clinical benefits of early ART that continue beyond 3 years—early ART is associated with lower longer-term mortality than standard ART—or the effect of early ART on disability and quality of life. Cost-effectiveness studies now need to be undertaken at different sites to determine whether these findings are generalizable but, for now, this cost-effectiveness study suggests that the new WHO guidelines for ART initiation can be cost-effective in resource-poor settings, information that should help policy-makers in developing countries allocate their limited resources.

**Additional Information:**

Please access these Web sites via the online version of this summary at http://dx.doi.org/10.1371/journal.pmed.1001095.

- Information is available from the US National Institute of Allergy and infectious diseases on HIV infection and AIDS
- HIV InSite has comprehensive information on all aspects of HIV/AIDS
- Information is available from Avert, an international AIDS charity on many aspects of HIV/AIDS, including information on the HIV/AIDS in the Caribbean, and on HIV/AIDS treatment and care (in English and Spanish)
- WHO provides information about universal access to AIDS treatment (in English, French and Spanish); its 2010 ART guidelines can be downloaded
- More information about the CIPRA HT-001 clinical trial is available
- Patient stories about living with HIV/AIDS are available through Avert and through the charity website Healthtalkonline
- More information about GHESKIO is available from Weill Cornell Global Health

## Introduction

In November 2009, the World Health Organization (WHO) changed its guidelines to recommend starting antiretroviral therapy (ART) in all HIV-infected patients when the CD4 cell count is less than 350 cells/mm^3^ rather than 200 cells/mm^3^ on the basis of results of the CIPRA HT-001 randomized trial conducted in Haiti, and a post hoc analysis nested within the SMART trial [1]–[3]. The panel that developed this recommendation “placed a high value on avoiding death, disease progression and HIV transmission over and above cost and feasibility” [1].

Implementing the new WHO recommendations will require countries to prioritize allocation of limited resources for ART medications, laboratory services, and clinic staff. At the end of 2009, 14.6 million people with HIV in low- and middle-income countries were considered in need of ART under the current WHO guidelines, and 5.3 million were receiving treatment [4]. Haiti has an estimated HIV prevalence of 2.2% [5]. At the end of 2009 HIV prevalence in Haiti was estimated at 120,000 individuals of whom 26,000, or 43% of those with CD4 cell count <350 cells/mm^3^, were receiving ART [6]. Evidence of cost-effectiveness will be a major factor in determining whether additional funding to initiate ART among patients who qualify under the new guidelines is an appropriate use of resources.

We conducted the first (to our knowledge) cost-effectiveness study of early versus deferred ART alongside a prospective randomized trial. CIPRA HT-001 demonstrated that among HIV-1 infected patients with a CD4 cell count between 200 and 350 cells/mm^3^, in a resource-poor setting after a median of 21 mo of follow-up, early ART reduces mortality by 75% compared with deferring ART until the CD4 cell count falls to 200 cells/mm^3^ or an AIDS-defining illness occurs [2]. We evaluated the costs incurred in each arm of the trial and compared the incremental cost of early ART to the mortality gain in order to determine the economic value of early ART after a maximum of 3 y with and without taking into account savings from excluding research-related laboratory tests.

## Methods

A randomized, open-label clinical trial of early versus standard ART in HIV-infected adults with no history of an AIDS-defining illness and a CD4 cell count between 200 and 350 cells/mm^3^ was conducted at the Center of the Haitian Group for the Study of Kaposi's Sarcoma and Opportunistic Infections (GHESKIO) in Port-au-Prince, Haiti. Subjects were excluded if they had a history of an AIDS-defining illness or had previously received ART [2]. The primary study end point was survival. The study was approved by the institutional review boards of GHESKIO, Weill Cornell Medical College (New York, New York, US) and Brigham and Women's Hospital (Boston, Massachusetts, US).

Between August 2005 and July 2008, 816 participants were seen monthly and received a package of medical services similar to that provided to HIV-infected patients at GHESKIO [7],[8]. The median (interquartile range) CD4 cell count at baseline was 280 (250–305) cells/mm^3^ in the early group and 282 (250–310) cells/mm^3^ in the standard group. Median (interquartile range) body mass index and hemoglobin at baseline were 21.3 (19.6–23.7) and 11.5 (10.3–12.6) g/dl, respectively, for the early group and 21.0 (9.2–23.4) and 11.4 (10.3–12.5) g/dl in the standard group; there were 28 participants with pulmonary tuberculosis in the early group and 15 in the standard group. Demographic characteristics were similar between groups. The median age at enrollment was 40 y, 58% were women, 39% had a secondary school or higher education, 63% were earning <US$100 per year, and 42% were living with a spouse or partner. The early group initiated lamivudine and zidovudine in a fixed-dose combination and efavirenz within 2 wk of enrollment. The standard group started the same first-line ART regimen when participants developed a single CD4 cell count measurement ≤200 cells/mm^3^ or an AIDS-defining illness. Among the 166 participants in the standard group who started first-line ART, the median (interquartile range) CD4 cell count at ART initiation was 160 (130–190) cells/mm^3^. In the event of treatment-limiting toxicity single-drug substitutions were allowed.

The trial protocol required that complete blood count (CBC), alanine aminotransferase (ALT), aspartate aminotransferase (AST), bilirubin, and creatinine tests be conducted every 3 mo for patients on ART. These tests are not routinely performed for nonstudy patients on ART at GHESKIO. The CD4 cell count was repeated for all participants every 6 mo or when clinically prompted and a CBC was obtained with every CD4 cell count, which is routine for patients on ART at GHESKIO.

At the second interim analysis the data safety monitoring board (DSMB) reviewed the trial data accumulated up to May 1, 2009, representing a median of 21-mo follow-up, and there were 23 deaths in the standard group and six in the early group (*p* = 0.0011 by the log-rank test). There were 37 patients (5%) lost to follow-up, 18 in the standard group and 19 in the early group. On the basis of a significant survival difference between groups, the DSMB recommended that the trial be stopped and all participants in the standard arm be provided with ART. There was also a significant 2-fold higher incidence of tuberculosis (TB) in the standard group (*n* = 36) versus the early group (*n* = 18) (*p* = 0.0125 by the log-rank test).

Table 1 summarizes the unit costs used to determine treatment costs. All costs are reported in 2009 US dollars; costs in local currency were converted at 40.47 Haitian gourdes = US$1 [9].

**Table 1:** Unit costs.

Cost	2009 US$
**ART and TB medications (monthly cost)**	
First-line ART (zidovudine/lamivudine and efavirenz)	18.86
Second-line ART (abacavir, tenofovir, and lopinavir/ritonavir)	79.39
TB treatment during initiation phase (isoniazid/rifampin, ethambutol, pyrazinamide)	9.73
TB treatment during maintenance phase (isoniazid/rifampin)	3.65
**Laboratory and other tests (cost per test)**	
CD4 cell count	30.00
Monitoring tests (ALT, AST, bilirubin, creatinine)	5.00
CBC	5.00
Tuberculosis acid fast bacilli smear	2.00
Tuberculosis culture	35.00
Chest radiograph^a^	35.00
**GHESKIO outpatient visits (cost per visit)**	
Scheduled^b^ or interim visit (MD cost only)	4.06
Sexually transmitted infection clinic	8.13
Prenatal clinic^c^	6.15
Family planning clinic (female/male patient)	1.76/0.99
Dermatology and other clinics	4.06
Social worker individual counseling	1.02
Social worker support group	0.30
Overhead (per MD visit)	6.27
**Outpatient nurse and pharmacist (cost per month)**	
Nurse before ART initiation	0.59
Pharmacist before ART initiation	0.27
Nurse after ART initiation	0.73
Pharmacist after ART initiation	0.72
**Other Support (cost per month)**	
Field worker	3.21
Nutritional supplementation	24.75
**Hospitalizations**	
Hospital services (bed, nursing, administration) per day	24.71
Hospital physician on day of admission	8.49
Hospital physician on other days	4.15
Study physician visit to hospitalized patients (includes travel time)	21.67
Specialist consultation	24.71
Major procedure^d^	185.32–1,235.48
**Patient and caregiver costs**	
Patient and caregiver time (per day)	1.75
Transportation (varies by residence zone)	0.94–7.17

a Cost of radiograph and reading by radiologist conducted off-site.

b Additional costs were incurred at initial study visit (US$5.42), study visits that included physician administration of an adherence and quality-of-life questionnaire (US$2.71) and physician visits that included initiation of ART for standard group (US$2.71).

c MD costs were reduced by one-half beyond 2 mo postdelivery.

d One dilation and curettage (US$185.32), one hospital birth (US$247.10), one appendectomy (US$420.06), one inguinal hernia repair (US$420.06), one leg repair (US$420.06), one cervical lymph node biopsy (US$494.19), one removal of uterine fibroids (US$1,235.48).

### Medications and Laboratory Tests

Medication use was documented in study records including start and stop dates for each drug. ART doses were specified, and for the remaining medications we used standard doses provided by the study staff. The cost of ART medications was set at the US President's Emergency Plan for AIDS Relief (PEPFAR) price in early 2009 [10]. The cost of TB medications was set at the International Dispensary Association (IDA; a nonprofit distributor) price plus 20% for importing and storage fees [11]. Other medications were purchased approximately 50% of the time from nonprofit distributors and 50% of the time from local distributors, so their costs were set at the average of IDA prices and local prices provided by the GHESKIO pharmacy.

All laboratory tests were performed on site and documented in the study records. The unit cost of each type of laboratory test had previously been calculated by GHESKIO accounting staff and included labor, reagents, and equipment.

### Labor and Overhead Costs

Labor costs were assigned to each visit date at GHESKIO on the basis of the type of services provided, the average duration of each service, and hourly labor costs using annual salaries and benefits for each job category. Study records were abstracted to determine dates of all clinic visits and missed visits.

Average duration of visits was measured using results from time and motion studies conducted previously for visits to HIV physicians (15 min), and to the sexually transmitted infection (30 min), family planning (18 min for female and 7 min for male patient), and counseling (20 min) units [7]. We added an additional 20 min at the first HIV physician visit and an additional 10 min at specified visits when the physicians also interviewed participants about adherence and quality of life using a standardized form. Physician time spent on follow-up activities such as chart documentation was assigned an additional 50% of the duration of each physician visit, as described previously [7]. The durations of other types of clinical visits were obtained from interviews. These marginal costs were used because physicians were also conducting research activities and were therefore working at full capacity.

Nurse, pharmacist, and fieldworker time could not be assigned accurately to specific dates and were applied to each patient study month, but reflected differences in nurse and pharmacist workload depending on whether the patient was on or off ART.

Overhead costs were identified from the GHESKIO budget for HIV care and were assigned to each participant as an additional cost for each HIV physician visit (Table 1).

### Other Outpatient Costs

Participants were referred to the on-site nutrition program to receive monthly allotments of beans, oil, flour, rice, and salt when the study staff felt it was clinically indicated. The start date for nutritional supplementation for each participant was abstracted from clinic records, and it was assumed that supplementation was provided for 6 mo from this date at a standard monthly cost calculated by the nutrition program.

Radiographs, other tests, and procedures were included only if there was documentation that they had occurred. The costs were actual payments made by GHESKIO or prices provided by local providers (Table 1). There were five visits to off-site specialists.

### Hospitalization Costs

Dates of hospital admission and discharge were recorded in the study database. We reviewed study and hospital records to determine resource use during the hospitalization, including intravenous fluids, supplies, medications, laboratory and radiographic tests, time of hospital physicians (using results of a time and motion study conducted at one hospital over 3 d), procedures, and specialist consultations. Complete hospital records were available for 66 hospitalizations and hospital discharge summaries only were available for 18 hospitalizations. GHESKIO costs were used for all medical supplies and labor rates. A daily cost for hospital services (bed, nursing, and administration) and costs for each of the seven major inpatient procedures that were recorded were from a survey of seven private hospitals in Port-au-Prince. The daily cost for hospital services is approximately US$25 (range US$12–US$52), which reflects the fact that in Haiti inpatient care is provided primarily by family caregivers (see below).

### Patient and Caregiver Costs

Patient time was 1 d for any outpatient visit or day in the hospital. Family caregiver time included 4 d for the family member to accompany patients initiating TB treatment and 1 d of family caregiver time for each day of hospitalization. The cost of patient and caregiver time was the minimum wage in Haiti at the time the study was conducted (US$1.75 per day) [12]. Transportation costs were calculated on the basis of residence zone [7].

### Analysis

Each of the resources used by each patient on each day was multiplied by the unit cost of that item, then summed to determine total costs for the arm. There were 528,623 total patient days observed. The costs for each arm were divided by the number of patients in the arm to determine the mean cost per patient for the duration of the trial; reporting costs for the entire trial period is typical when cost-effectiveness analyses are conducted alongside clinical trials [13],[14]. Differences between arms in costs for the duration of the trial were compared using Wilcoxon rank-sum tests to account for potential skewness in cost data. The mean survival time was estimated by the area under the Kaplan-Meier curve and the mean cost was estimated by the nonparametric method of Zhao and Tian in order to account for censoring [15],[16]. Censoring occurs because patients were enrolled on different dates but observed only until death, loss to follow-up, or the stopping date of May 1, 2009. Analyses of mean cost must be properly adjusted for censoring because, although the time of events (e.g., mortality) and the time of censoring are independent, their cost counterparts are not (informative censoring) [15],[16]. Due to this nonstandard censoring mechanism, traditional methods such as sample average and Kaplan-Meier estimation are inappropriate for cost data [17]. Undiscounted, observed costs were used for comparisons between arms. Cost-effectiveness ratios used censoring-adjusted life expectancy and, following typical but not universal practice, costs and life expectancy were both discounted at a 3% annual rate [18]–[20]. Cost-effectiveness ratios were calculated with a maximum of 3 y of follow-up because of unreliable cost estimates beyond 3 y. Cost-effectiveness ratios were calculated as the incremental discounted cost of the early arm versus the standard arm divided by the incremental discounted life expectancy of the early arm versus the standard arm. Hence these ratios should be interpreted as comparing the cost-effectiveness of early versus standard ART only during the trial observation period, up to a maximum of 3 y. Confidence intervals for the cost-effectiveness ratios were calculated using Fieller's theorem, which is used to calculate confidence intervals for the ratio of two means [16],[21],[22]. Nonparametric bootstrapping was used to construct cost-effectiveness acceptability curves [21].

Cost-effectiveness ratios were calculated with and without research-related laboratory monitoring tests for ART toxicities. We believe findings excluding research-related tests are more policy relevant because they reflect current clinical practice and there is evidence that the availability of these test results does not change clinical management in resource-limited settings. The authors of the multicenter randomized DART study conducted in Africa concluded that “ART can be safely delivered without routine laboratory monitoring for toxic effects” [23]. A GHESKIO study showed that the utility of routine laboratory monitoring is minimal, rarely leading to a change in medications [24]. Therefore, ALT, AST, bilirubin, creatinine, and CBC tests are not performed routinely for ART patients at GHESKIO. To identify research-related laboratory tests, we first excluded tests considered to be clinically indicated because they were (1) conducted at an interim (nonscheduled) visit; (2) conducted prior to ART initiation; or (3) CBC tests associated with a CD4 cell count (a CBC is required for interpretation of the CD4 count). On the basis of the DART study findings, we estimated that of the remaining tests conducted at scheduled study visits 3.1% of CBC tests and 2.5% of ALT, AST, bilirubin, and creatinine tests would have been clinically indicated and ordered by the study physician in the absence of the protocol requirement [23]. Research-related CBC, ALT, AST, bilirubin, and creatinine tests were therefore calculated as 84.1% and 37.8% of the total number of these tests conducted in the early and standard groups, respectively.

We performed sensitivity analyses on research-related laboratory testing and ART cost inputs. For laboratory testing, we considered a scenario that excluded research-related testing but where CBC testing was performed routinely every 3 mo for all patients on zidovudine, resulting in 79% and 36% of all tests being considered research related in the early and standard groups, respectively. This is a conservative assumption because hemoglobin monitoring every 3 mo is recommended in WHO guidelines only for patients with low body weight or a low CD4 cell count, and anemia can be detected with a less expensive hematocrit test [25]. In ART cost-sensitivity analyses, we (1) reduced the cost of efavirenz by 50% to reflect the cost in Haiti at the beginning of 2010 versus at the time of the study, and (2) substituted tenofovir for zidovudine in first-line regimens using the current cost of tenofovir and adding the cost of creatinine tests every 6 mo [25], and (3) varied the cost of second-line ART by 50%. Two-sided hypotheses/tests were adopted for all statistical inferences and statistical analyses were performed using SAS 9.2 (SAS Institutes).

## Results

Mean total treatment costs per patient for the duration of the study and per year in the study were US$1,381 (US$810 per year) for early ART and US$1,033 (US$631 per year) for standard ART, a cost difference of US$348 (US$179 per year) (*p*<0.0001 for study duration). After excluding research-related laboratory tests, per patient costs were US$1,158 (US$679 per year) and US$979 (US$604 per year), respectively, a difference of US$179 (US$75 per year) (*p*<0.0001 for study duration). Outpatient treatment costs are reported in Table 2. They were 92% of the total treatment cost in the early group and 86% in the standard group (mean costs of US$1,271 and US$888 per patient, respectively; *p*<0.0001) (Table 2). The mean cost per patient of ART during the study was higher in the early treatment group than the standard treatment group (US$398 versus US$81; *p*<0.0001) and the early group had higher per patient nurse and pharmacist costs during the study (mean US$33 versus US$21; *p*<0.0001). Participants in the early group had fewer HIV physician visits (mean 13.2 versus 14.6 visits; *p* = 0.0052), lower HIV physician visit costs (mean US$78 versus US$87; *p* = 0^.^0006), and lower costs of non-ART medications (mean US$58 versus US$71; *p* = 0.0002) during the study.

**Table 2:** Outpatient cost per person during the trial (2009 US$).

Cost	Early Treatment Mean (95% CI) *n* = 408		Standard Treatment Mean (95% CI) *n* = 408		*p*-Value*
	Trial Period	Yearly	Trial Period	Yearly	
**Medications**					
Antiretroviral therapy	398 (380–416)	218 (213–222)	81 (67–95)	38 (32–44)	<0.0001
TB medications	7 (5–9)	5 (3–6)	7 (5–9)	5 (3–7)	0.4708
Other medications	51 (48–55)	30 (28–31)	64 (59–69)	38 (35–40)	<0.0001
Total cost of medications	456 (436–476)	252 (247–257)	152 (136–169)	80 (74–87)	<0.0001
**Laboratory tests**					
CD4 cell count	122 (114–130)	64 (61–68)	171 (160–183)	97 (91–102)	<0.0001
Liver function tests (ALT, AST, bilirubin)	157 (152–163)	92 (89–95)	63 (55–72)	34 (30–39)	<0.0001
Creatinine	53 (51–55)	31 (30–32)	21 (19–24)	12 (10–13)	<0.0001
CBC	56 (54–58)	32 (32–33)	41 (38–45)	23 (21–25)	<0.0001
Other laboratory tests	39 (34–43)	23 (20–26)	50 (44–56)	30 (26–34)	0.0237
Total cost of laboratory tests	427 (407–446)	243 (233–252)	347 (320–374)	196 (181–211)	<0.0001
**Other tests**					
Chest radiographs	14 (11–16)	8 (6–11)	20 (17–24)	13 (10–15)	0.0214
Other noninvasive tests	2 (1–3)	1 (1–2)	3 (1–5)	3 (0–6)	0.8334
Total cost of other tests	15 (13–18)	10 (7–13)	23 (19–28)	15 (11–20)	0.0099
**Outpatient care**					
HIV physician visits	78 (75–81)	47 (45–49)	87 (84–91)	55 (53–57)	0.0006
Nurse and pharmacist	33 (32–34)	18 (18–18)	21 (20–22)	12 (12–12)	<0.0001
Prenatal care (including home births or miscarriages)	2 (0–3)	1 (0–1)	2 (0–3)	1 (0–1)	0.6743
Other outpatient visits	2 (2–3)	1 (1–2)	3 (2–4)	2 (1–2)	0.6040
Field workers	72 (70–75)	40 (40–40)	69 (66–72)	40 (40–41)	0.1277
Overhead^a^	83 (79–86)	48 (46–50)	92 (87–96)	56 (53–58)	0.0052
Total cost of outpatient care	270 (259–280)	156 (151–160)	274 (262–286)	166 (161–170)	0.9055
**Nutrition Costs**	103 (97–109)	61 (56–65)	90 (84–97)	61 (55–68)	0.0057
**Total outpatient health system cost**	1,271 (1,219–1,322)	721 (703–739)	888 (832–943)	519 (492–545)	<0.0001
**Patient and caregiver costs**					
Time	26 (25–27)	15 (15–16)	29 (28–31)	18 (17–19)	0.0026
Transportation	36 (33–39)	22 (20–24)	47 (42–51)	28 (26–31)	0.0111
Total patient and caregiver costs	62 (59–66)	38 (36–40)	76 (70–81)	46 (43–49)	0.0072
**Total outpatient cost**	1,333 (1,280–1,386)	759 (740–778)	963 (904–1,023)	565 (537–593)	<0.0001

a Overhead costs include administrative salaries and benefits, monitoring and evaluation, building and equipment cleaning and maintenance, electricity, transportation and storage of medications, security services, computers, furniture, office supplies, and telephone.

*p-Values are for trial period and were computed by Wilcoxon rank sum tests.

In the early group, 4,585 CBC tests were performed (57% research-related) versus 3,380 in the standard group (17% research-related), and 12,841 ALT and AST, bilirubin, and creatinine tests were performed (91% research-related) versus 5,183 in the standard arm (46% research-related). Mean costs per patient during the study for these tests in the early group were higher including research-related tests (US$266 versus US$126; *p*<0.0001), and lower excluding research-related tests (US$43 versus US$72; *p*<0.0001). Fewer CD4 cell counts were conducted in the early group (1,659 versus 2,330) leading to a lower mean cost per patient during the study (US$122 versus US$171; *p*<0.0001). The early group also had significantly lower costs for other laboratory tests and chest radiographs. The total per patient cost for non-ART medications, CD4 cell counts, clinically indicated tests, and radiographs during the study was about 30% lower in the early group (US$275 versus US$384; *p*<0.0001).

Hospitalization comprised 3% of the total cost for the early group and 6% for the standard group (mean costs per patient during the study of US$44 and US$63 respectively; *p* = 0.2388). There were 84 hospitalizations during the study, 36 in the early group and 48 in the standard group; a total of 28 patients were hospitalized in the early group and 37 patients in the standard group. One private hospital in Port-au-Prince was the primary site for hospital referrals, accounting for 63 hospitalizations representing 82% of days that patients spent in the hospital. Reasons for hospitalizations included TB (seven early group, 11 standard group), gastroenteritis (five early group, 11 standard group), anemia (six early group, six standard group), pneumonia (one early group, four standard group), and other indications (17 early group, 16 standard group). The median length of stay per hospitalization was shorter for the early treatment group (7.5 d versus 10.0 d), but costs per hospitalization were similar between the two groups (US$543 for the early treatment group versus US$590 for the standard treatment group; *p* = 0.6033) (Table 3). Patient and caregiver costs incurred as either an outpatient or an inpatient composed 5% of the total per patient cost for the early group and 8% of the total per patient cost for the standard group (mean US$66 and US$82, respectively; *p* = 0.0059).

**Table 3:** Inpatient cost per hospitalization (2009 US$).

Cost	Early Treatment Mean (95% CI) *n* = 36	Standard Treatment Mean (95% CI) *n* = 48	*p*-Value*
**Medical Products**			
Medications	29 (0–59)	32 (8–56)	0.2903
Intravenous fluids, blood transfusions, oxygen	27 (15–38)	22 (15–28)	0.2328
Total cost of medical products	56 (24–88)	54 (27–80)	0.8247
**Tests and procedures**			
Laboratory tests	11 (4–17)	15 (8–23)	0.4861
Radiographs and other noninvasive tests	13 (3–24)	15 (5–26)	0.5971
Procedures^a^	63 (0–139)	25 (0–53)	0.9168
Total cost of tests and procedures	87 (9–165)	56 (25–86)	0.5660
**Physician visits**	58 (33–82)	71 (48–95)	0.3922
**Hospital services (administration, nursing, bed)**	296 (155–437)	355 (255–455)	0.2713
**Total health system cost**	497 (306–688)	536 (393–679)	0.5907
**Patient and caregiver costs**			
Time	41 (22–61)	50 (36–64)	0.2713
Transportation	5 (4–6)	4 (3–5)	0.3252
Total patient and caregiver costs	46 (26–66)	54 (40–68)	0.2923
**Total Inpatient Cost**	543 (334–752)	590 (434–747)	0.6033

a Includes major procedures and other procedures such as thoracentesis or insertion of nasogastric tube costing <US$10.

*p-Values were computed by Wilcoxon rank sum tests.

**Figure 1:**
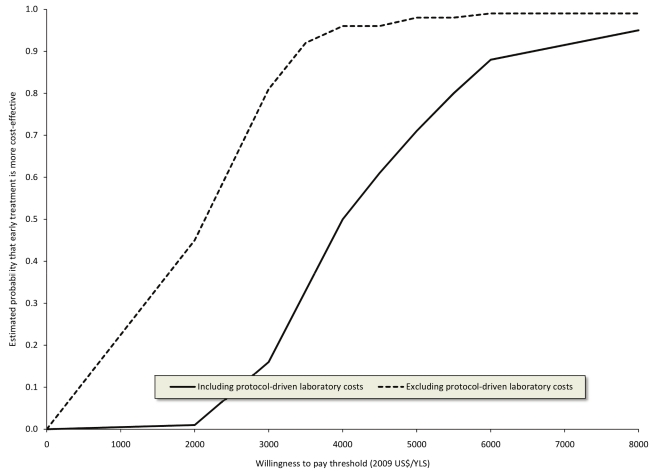
Cost-effectiveness acceptability curves for earlier initiation of ART after a maximum of 3 y including and excluding protocol-driven costs. Curves were constructed from 100 bootstrap simulations including protocol-driven costs and 100 bootstrap simulations excluding protocol-driven costs.

The discounted mean survival time after a maximum of 3 y after adjusting for censoring was 1,035 d (2.84 y) in the early group and 998 d (2.73 y) in the standard group. The discounted cost of treatment after adjusting for censoring was US$1,965 per patient for the early group and US$1,555 per patient for the standard group including research-related laboratory tests (an incremental difference of US$410); excluding these tests the costs were US$1,660 and US$1,448 per patient respectively (an incremental difference of US$212). The cost-effectiveness ratio for early versus standard ART after a maximum of 3 y was US$3,975/year of life saved (YLS) (95% CI US$2,129/YLS–US$9,979/YLS) including research-related tests, and US$2,050/YLS excluding research-related tests (95% CI US$722/YLS–US$5,537/YLS) (Table 4). Cost-effectiveness acceptability curves were consistent with these mean and 95% CI results (Figure 1).

**Table 4:** Cost-effectiveness of earlier initiation of ART after a maximum of 3 y.

Strategy	Mean (95% CI) Cost of Treatment (2009 US$)	Mean (95% CI) Days Survival Time (d)	Mean (95% CI) Incremental Cost-Effectiveness Ratio (2009 US$/YLS)^a^
**Including protocol-driven laboratory costs**			
Standard treatment group	1,555 (1,424–1,686)	998 (978–1,018)	
Early treatment group	1,965 (1,908–2,022)	1,035 (1,025–1,045)	3,975 (2,129–9,979)
**Excluding protocol-driven laboratory tests**			
Standard treatment group	1,448 (1,332–1,564)	998 (978–1018)	
Early treatment group	1,660 (1,601–1,719)	1,035 (1,025–1,045)	2,050 (722–5,537)
**Sensitivity analyses including protocol-driven laboratory costs**			
**Current efavirenz cost** b			
Standard treatment group	1,508 (1,383–1,633)	998 (978–1,018)	
Early treatment group	1,817 (1,756–1,878)	1,035 (1,025–1,045)	3,904 (1,423–7,707)
**Substituting tenofovir for zidovudine with creatinine testing twice annually, current tenofovir cost**			
Standard treatment group	1,565 (1,432–1,698)	998 (978–1,018)	
Early treatment group	2,002 (1,943–2,061)	1,035 (1,025–1,045)	5,289 (2,307–15,593)
**50% lower cost of second-line ART**			
Standard treatment group	1,555 (1,424–1,686)	998 (978–1,018)	
Early treatment group	1,959 (1,902–2,016)	1,035 (1,025–1,045)	3,918 (2,091–9,841)
**50% higher cost of second-line ART**			
Standard treatment group	1,555 (1,424–1,686)	998 (978–1,018)	
Early treatment group	1,971 (1,912–2,030)	1,035 (1,025–1,045)	4,982 (2,166–10,118)
**Sensitivity analyses excluding protocol-driven laboratory costs**			
**Quarterly CBC testing while on zidovudine/lamivudine**			
Standard treatment group	1,456 (1,338–1,574)	998 (978–1,018)	
Early treatment group	1,688 (1,629–1,747)	1,035 (1,025–1,045)	2,246 (876–5,978)
**Current efavirenz cost** b **and 50%lower cost of second-line ART**			
Standard treatment group	1,401 (1,289–1,513)	998 (978–1,018)	
Early treatment group	1,506 (1,447–1,565)	1,035 (1,025–1,045)	1,013 (cost-saving–3,326)

All results are rounded to the nearest integer.

a Confidence intervals for the cost-effectiveness ratios were calculated using the Fieller's theorem for the ratio statistics [16],[21],[22].

b 50% reduction in cost of efavirenz.

When we assumed routine CBC testing was performed every 3 mo for all patients on zidovudine the cost-effectiveness ratio excluding research-related tests was US$2,264/YLS (95% CI US$876/YLS–US$5,978/YLS). The cost-effectiveness ratios including research-related tests were slightly lower using the current efavirenz cost and after reducing the second-line therapy cost; these ratios were higher when tenofovir was substituted for zidovudine and after increasing the second-line therapy cost. In the best case, (excluding research-related tests, using the current efavirenz cost and reducing the second-line therapy cost) the cost-effectiveness ratio was US$1,013/LY (95% CI cost-saving US$3,326/YLS) (Table 4).

## Discussion

We evaluated the costs incurred in each arm of the CIPRA HT-001 trial and compared the incremental cost and survival benefit of early versus standard ART in order to determine the cost-effectiveness of early ART over a maximum 3-y time horizon. Higher ART and associated nursing and pharmacist costs in the early ART group were partially offset by higher costs for HIV physician visits, other medications, CD4 cell counts, clinically indicated laboratory tests, and radiographs in the standard group.

These are the only data, to our knowledge, comparing the cost of early versus standard ART using data from a randomized trial that compared these two strategies. HIV treatment protocols, laboratory tests, and medication costs are similar to those in other resource-poor settings, particularly programs funded in part by US President's Emergency Plan for AIDS Relief (PEPFAR). Our findings are also generalizable to nontrial settings, as the patients in the CIPRA HT-001 trial received nearly identical medical services as nonstudy patients, with similar frequencies of physician and nurse contacts compared to those previously described for usual care at GHESKIO [7].

The WHO-CHOosing Interventions that are Cost Effective (CHOICE) Working Group designates interventions as cost-effective if the cost per disability-adjusted life year (DALY) averted is less than three times the country's per capita gross domestic product (GDP) and very cost-effective if the cost per DALY is less than one times the country's GDP per capita [26]. Between and even below these thresholds, each country needs to consider what interventions to fund and how to obtain additional financial support to expand coverage. Although our analysis computes cost-effectiveness ratios in terms of YLS rather than using DALYs, the WHO threshold provides general guidance that has been used in other studies using YLS [27],[28]. In Haiti this threshold was US$2,355/YLS in 2009 [29]. The cost-effectiveness ratio of early versus standard ART was above this threshold if research-related tests were included (US$3,975/YLS), but below the threshold if research-related tests were excluded (US$2,050/YLS). More aggressive monitoring for anemia on zidovudine slightly increased the cost-effectiveness ratio, while substituting tenofovir for zidovudine or using a higher cost second-line ART regimen resulted in more substantial increases in the cost-effectiveness ratio. Recent trends, however, indicate that costs for tenofovir and second-line regimens are declining [30].

These cost-effectiveness ratios are highly conservative (biased against early ART), because they do not include the clinical benefits of earlier treatment that would continue beyond the maximum 3-y time horizon of our study. The median CD4 cell count at ART initiation was 280 cells/mm^3^ in the early group and 166 cells/mm^3^ in the standard group. Multiple studies have demonstrated that higher baseline CD4 cell counts are associated with improvements in immunologic recovery and lower long-term mortality on ART [31]–[33]. In contrast, a significant proportion of patients who defer ART until the CD4 cell count drops below 200 cells/mm^3^ will fail to achieve a normal CD4 cell count and will experience a higher rate of morbidity and mortality from both AIDS- and non-AIDS–related diseases, even after 7 to 10 y of otherwise effective therapy [31],[34]–[36]. Additional benefits of earlier treatment that were not measured include averted cases of TB among contacts of the participants associated with the lower rate of active TB infection in the early group, and the reduction in HIV transmission with earlier initiation of ART [37],[38].

Our study results are important for low- and middle-income countries to consider as they decide whether to adopt the new WHO guidelines. ART costs in the standard group were US$81 per person over the median follow-up time of 21 mo because, even through these participants did not initiate ART early, 39% of them subsequently had a CD4 cell count measurement ≤200 cells/mm^3^ or developed an AIDS-defining illness and initiated ART. ART costs in the early group were US$317 higher (US$398 per person), but these incremental ART costs were partly offset by savings in the cost of non-ART medications, CD4 cell counts, clinically indicated tests, and radiographs. In countries that have access to similar ART prices but have higher labor rates and a more developed hospital infrastructure, care cost savings might be greater.

To our knowledge, this is the first cost-effectiveness study of early versus deferred ART eligibility thresholds conducted alongside a prospective randomized trial. The trial was designed to minimize loss to follow-up in order to obtain valid study endpoints in both arms, and therefore does not take into account recent observations from South Africa that HIV-infected patients with a CD4 cell count above a threshold for ART initiation are much more likely to be lost to follow-up than those who can initiate treatment immediately [39],[40]. Patient adherence to medications and physician adherence to guidelines in a clinical trial setting may also differ from nontrial settings [41]. Nevertheless, our findings are similar to published results from a computer simulation model of HIV disease in the medium term that conducted sensitivity analyses addressing these issues. Walensky et al. found that in South Africa, the incremental cost-effectiveness ratio for initiating ART at a threshold of 350 cells/mm^3^ was US$2,400/YLS compared with initiating ART at 250 cells/mm^3^, when measured over a 5-y time horizon [27]. In a study conducted in Morocco, initiating ART >200 cells/mm^3^ had a cost-effectiveness ratio that was nearly three times GDP per capita when measured over a 5-y time horizon [42]. Although longer follow-up is not feasible in a clinical trial, several computer simulation results show that the cost-effectiveness of earlier ART is lower with a lifetime perspective: US$1,200/YLS and US$616/QALY in South Africa and US$1,530/YLS in India [27],[28],[43]. The long-term cost-effectiveness of early versus standard ART in this study will depend on whether the early group continues to have a survival benefit after standard group patients have initiated ART and whether there are any differences in second-line ART initiation rates between the two groups in the future; these data are currently being collected.

Beyond the absence of long-term follow-up, our study has additional limitations. We report years of life saved because there are no data on disability or quality-of-life measures for patients with early HIV disease in Haiti. If we had, earlier treatment would likely have been even more cost-effective, because the quality of life benefit of avoiding the 18 additional cases of TB that occurred in the standard group would outweigh the small number of additional drug-related adverse events observed in the early group [2]. The cost-effectiveness ratio was lower when we excluded research-related tests. We are confident on the basis of results of a large randomized trial [23] and GHESKIO clinic data [24] that clinical outcomes would have been unchanged in the absence of these tests. Although our study was conducted at one site, many of our findings will be generalizable to other resource-poor settings because we use similar treatment protocols. Our study only addresses relatively short-term costs, i.e., up to a maximum of 3 y. Follow-up data on patient survival, new treatment protocols (such as introduction of targeted HIV viral load monitoring) [25], and costs will allow us to address long-term economic outcomes.

There are substantial budget and logistical constraints to implementing earlier treatment, including ensuring priority access to ART for the sickest patients and not diverting resources away from identifying these patients and retaining them in care. Furthermore, the results of cost-effectiveness analyses should only be one element in the priority setting process in the face of budget constraints. On the other hand, the CIPRA HT-001 trial was stopped because earlier treatment substantially decreased mortality [2], and our economic analysis indicates that it can be cost-effective in resource-poor settings.

Initiating ART in HIV-infected adults with a CD4 cell count between 200 and 350 cells/mm^3^ in Haiti is cost-effective after excluding laboratory monitoring without clinical benefit. Financial and operational resources should be prioritized so that resource-poor countries are able to implement the new WHO guidelines, which recommend treatment for all HIV-infected patients with CD4 cell counts <350 cells/mm^3^.

## Abbreviations

ALT: alanine aminotransferase
ART: antiretroviral therapy
AST: aspartate aminotransferase
CBC: complete blood count
GDP: gross domestic product
GHESKIO: Groupe Haitien d'Etude du Sarcome de Kaposi et des Infections Opportunistes
TB: tuberculosis
WHO: World Health Organization
YLS: year of life saved
